# Biodegradable Water-Soluble Matrix for Immobilization of Biocidal 4-Hexylresorcinol

**DOI:** 10.3390/ijms241914717

**Published:** 2023-09-28

**Authors:** Olga A. Novoskoltseva, Ekaterina A. Litmanovich, Nataliya G. Loiko, Yury A. Nikolaev, Alexander A. Yaroslavov

**Affiliations:** 1 Faculty of Chemistry, Lomonosov Moscow State University, 119991 Moscow, Russia; nsn07@yandex.ru (O.A.N.); elitmanovich@yandex.ru (E.A.L.); 2 Department of Microbiology, Federal Research Center “Fundamentals of Biotechnology” RAS, 119071 Moscow, Russia; loikonat@mail.ru (N.G.L.); nikolaevya@mail.ru (Y.A.N.)

**Keywords:** polysaccharide, interpolyelectrolyte complex, 4-hexylresorcinol, composite coating, biocidal activity

## Abstract

Biocidal coatings have been used in biomedicine, cosmetology and the food industry. In this article, the coatings are described as being composed of non-stoichiometric polycomplexes, products of electrostatic coupling of two commercial biodegradable ionic polymers, anionic sodium alginate and cationic quaternized hydroxyethyl cellulose ethoxylate. Non-stoichiometric polycomplexes with a 5-fold excess of the cationic polymer were used for immobilizing hydrophobic biocidal 4-hexylresorcinol (HR). Being dispersed in water, the polycomplex particles were capable of absorbing a tenfold excess of HR in relation to the polycation. After deposition onto the plastic surface and drying, the aqueous polycomplex–HR composite formulation forms a transparent homogeneous coating, which swells slightly in water. The interpolyelectrolyte complex (IPEC) is substantially non-toxic. The incorporation of HR in the IPEC imparts antimicrobial activity to the resulting composite, in both aqueous solutions and coatings, against Gram-negative and Gram-positive bacteria and yeast. The polysaccharide-based polycomplexes with embedded HR are promising for the fabrication of biocidal films and coatings.

## 1. Introduction

Environmental protection has become one of the most pressing issues over the past few decades [1,2,3]. Among other things, this trend involves replacing, where possible, synthetic polymers with their natural counterparts [4]. Polysaccharides are renewable and environmentally friendly biomaterials characterized by high bioavailability, biocompatibility and biodegradability [5,6]. Polysaccharides can be easily modified, and this quality improves operational properties of polymer materials and expands their application areas. Sodium alginate (Alg), a widely used anionic polysaccharide, finds numerous applications in biomedicine for drug delivery [7,8,9], the food industry for packaging [10,11,12,13,14], tissue engineering to accelerate wound healing and cell transplantation [7,8,15,16,17,18], cosmetics as water-retaining agents [19,20,21,22], etc. 

In order to stabilize Alg constructs—coatings, films and fibers—when surrounded by water, the polymer is converted into an ionotropic gel via crosslinking with a bivalent metal salt like CaCl_2_ [9,23,24], mixed Alg with hydrophobic polymers, e.g., cellulose nanofibers [25], or low-molecular-weight compounds [10,26], including those with bactericidal properties [27,28], such as curcumin, kaempferol and cinnamon essential oils [29,30,31]. 

4-Hexylresorcinol (HR) is an effective antiseptic capable of reducing/preventing pain as well as skin irritation and inflammation [32,33,34,35]. Due to the long-chain alkyl radical, HR can be incorporated into cell membranes, disrupting their integrity and increasing their permeability, thus affecting many vital processes such as energy production and nutrient transport. HR can react with proteins and change their structure, influencing metabolic pathways [36,37,38]. Pronounced biocidal properties of HR are used when preparing bactericidal soaps, antibacterial oral formulations, personal care products, skin and wound disinfectants and preoperative skin preparations [32,33,34,35,39,40,41]. HR can be combined with various biomaterials, including bone substitutes, medical silk threads and dental implants [40,41,42]. HR has antioxidant properties and is approved as a food additive [43]. 

At the same time, the hydrophobic tail makes HR poorly soluble in water. For this reason, HR is usually dissolved in organic solvents such as ethanol and dimethyl sulfoxide [42,44]. However, organic solvents and ionic polysaccharides are not compatible, which complicates work with Alg–HR mixtures and the preparation of biocidal biodegradable Alg–HR composites. 

In the current article, we describe the immobilization of HR in interpolyelectrolyte complexes (IPECs), products of electrostatic interactions between two oppositely charged ionic polymers: anionic Alg and cationic quaternized hydroxyethyl cellulose ethoxylate, Polyquaternium 10 (Pq10). Biodegradable Pq10 is included in shampoos and other hair care products, detergents and skin care formulations, as well as multipurpose solutions for the care of soft contact lenses [45,46]. For the immobilization of hydrophobic HR, non-stoichiometric IPECs were used, in which only 20 mol% of cationic Pq10 groups was electrostatically complexed with anionic Alg groups. Such IPECs were actually block-copolymers with hydrophobic fragments composed of mutually neutralized groups of both polymers and hydrophilic sequences of free cationic Pq10 groups. The former ensured the binding of hydrophobic HR, and the latter ensured the solubility of the entire structure in water. We examined the Alg-to-Pq10 complexation, the stability of the resulting IPEC in water–salt solutions, the incorporation of HR into the IPEC matrix, the formation of composite IPEC–HR coatings and the biocidal properties of IPEC–HR constructs in solution and in the coatings. As a result, a protocol is described which allows for preparing water-compatible polymer–HR composites with a high content of biocidal HR. These composites can form biocidal coatings that are stable when surrounded by water.

## 2. Results and Discussion

### 2.1. Electrostatic Interaction between Polysaccharides

**Figure 1:**
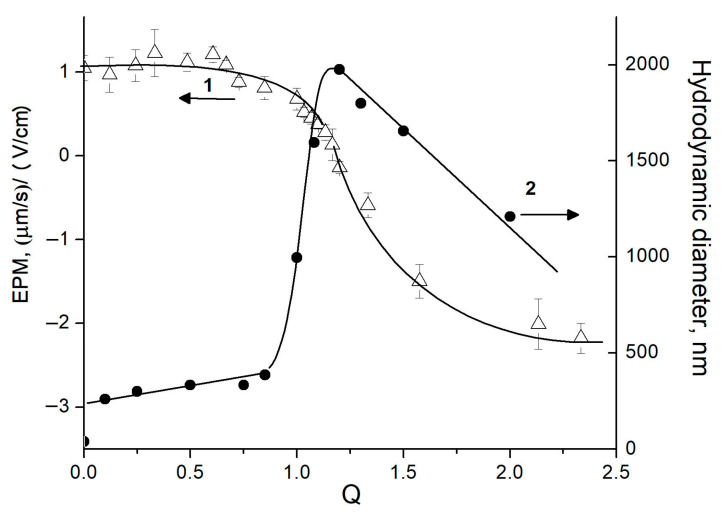
Electrophoretic mobility (1) and hydrodynamic diameter (2) of IPEC particles vs. Q ratio. [Pq10] = 5 × 10^–4^ M, 10^–2^ M pH 7 phosphate buffer; 25 °C.

Anionic Alg (*Mw* 79 kDa) and cationic Pq10 (*Mw* 540 kDa) were each dissolved in pH 7 phosphate buffer and the solutions were mixed at varied molar ratios of anionic Alg groups to cationic Pq10 groups, Q = [Alg]/[Pq10]. The addition of Alg solution to a Pq10 solution altered the polycation charge; this was detected via measuring the electrophoretic mobility (EPM) of Pq10 macromolecules, the parameter associated with their charge (Figure 1, curve 1). These results evidenced an electrostatic Alg-to-Pq10 complexation and formation of Alg-Pq10 IPEC. As shown earlier [47,48], the electrostatic interaction of oppositely charged multi-ions is accompanied by the quantitative binding of both polymers into IPEC. It follows from here that the composition of the resulting IPEC is equal to the composition of the initial polyanion/polycation mixture [47,49]. Obviously, the same is true for the IPEC formation from Alg and Pq10, whereby each of which has a high molecular mass.

A complete neutralization of the Pq10 charge by the Alg charge (EPM = 0) was achieved at Q = [Alg]/[Pq10] = 1.2 (Figure 1, curve 1). Taking into account (1) the quaternized amino-groups in the Pq10, which gave the maximum positive charge to the Pq10 chains in the pH 7 buffer, and (2) the quantitative Alg-to-Pq10 complexation, the molar degree of the anionic Alg groups electrostatically complexed with the cationic Pq10 groups was estimated: *ω* = (1/1.2) × 100% = 83%. A residual 17% of anionic ALG groups remained protonated in the pH 7 buffer solution and did not interact with cationic Pq10. A further increase in Q over 1.2 resulted in negatively charged IPEC particles due to the adsorption of Alg excess.

In parallel, the size of the IPEC particles in the system was detected (Figure 1, curve 2). In the Q < 0.85 region, particles 250–350 nm in diameter were formed, whose stability was due to abundant positive charges of free Pq10 groups. The mutual neutralization of Alg and Pq10 charges resulted in the maximum particle size. 

### 2.2. Incorporation of HR in Polycomplex Matrix

Alg is incompatible with the organic solvent used to prepare HR solutions [50]. For example, the mixing of Alg aqueous solution with HR ethanol solution is accompanied by the precipitation of Alg and formation of coarse dispersions. In order to immobilize HR, the Q = 0.2 Alg-Pq10 polycomplex was taken. The high positive charge ensured the aggregation stability of IPEC in the aqueous buffer solution. The immobilization was performed via the addition of HR ethanol solution to the freshly prepared, slightly opalescent Alg-Pq10 buffer solution. 

**Figure 2:**
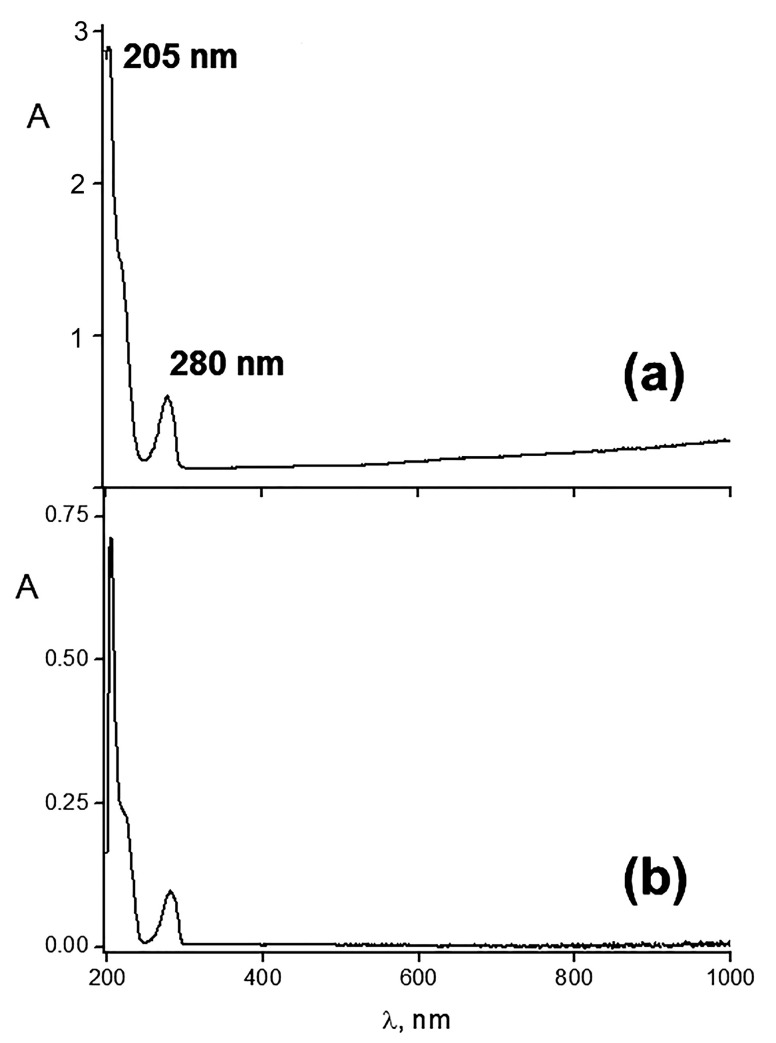
Electronic absorption spectra of (**a**) 10^–3^ M HR ethanol solution and (**b**) IPEC–HR composite solution in a 10^–2^ M pH 7 phosphate buffer/ethanol mixture (98/2 *v*/*v*), Q = 0.2; [Pq10] = 5 × 10^–3^ M; [HR] = 10^–3^ M.

Figure 2a shows an electronic spectrum of HR ethanol solution with two characteristic peaks at 205 and 280 nm that is in agreement with the literature data [51]. The spectrum of the IPEC–HR composite (Figure 2b) showed the same profile with the two peaks at 205 and 280 nm, which evidenced the incorporation of HR into the IPEC matrix; a calculation gave a nearly equimolar HR/Alg ratio in the IPEC–HR composite (see legend to Figure 2b). However, the Q = 0.2 IPEC was able to incorporate an additional amount of HR, up to a 10-fold excess in comparison to the Alg content, which was at [HR]/[Alg] = 10 molar ratio.

A reason for the extremely high capacity of water-soluble Q = 0.2 IPEC to HR is in the structure of the polycomplex species. The latter are block-copolymers with hydrophobic fragments represented by mutually neutralized Alg and Pq10 groups and free cationic Pq10 sequences. When surrounded by water, an IPEC particle acquires the structure with the hydrophobic core and cationic shell, capable of absorbing a significant amount of hydrophobic low-molecular-weight compounds like HR. This mechanism was earlier described for the interaction of IPECs with hydrophobic compounds [52,53]. The IPEC core acts as a variable volume reservoir whose size and capacity can greatly change.

An aqueous solution of IPEC–HR composite with an [HR]/[Alg] = 10 ratio had the maximum viscosity due to hydrogen bonds which stabilized the composite network [54].

### 2.3. Formation of Composite Biocidal Coatings

The next step was to use IPEC formulations for preparing biocidal coatings on the solid surfaces. These coatings (films) are promising for the antimicrobial protection of food products [55,56]. Individual polysaccharides, both anionic and cationic, are soluble in water and require modification, for example via cross-linking with divalent cations [23,24]. This procedure results in the polymer network which is insoluble in water but only slightly swells in it.

**Figure 3:**
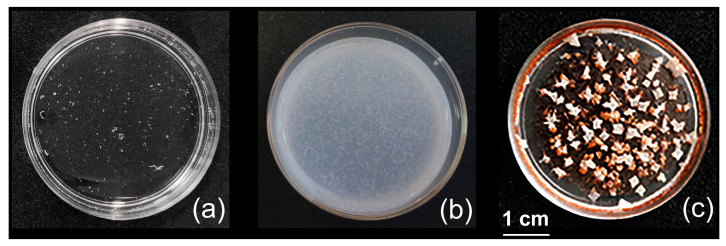
Photos of coatings (**a**) from IPEC solution, Q = 0.2, [Pq10] = 2 × 10^–2^ M, 10^–2^ M pH 7 phosphate buffer; (**b**) IPEC–HR composite solution in a 10^–2^ M pH 7 phosphate buffer/ethanol mixture (98/2 *v*/*v*), Q = 0.2; [Pq10] = 3.3 × 10^–3^ M; [HR] = 5 × 10^–2^ M; (**c**) from sample (**b**) after a 4 N NaCl solution treatment. The scale bar applies to each subfigure.

The addition of the Q = 0.2 IPEC aqueous solution in a plastic Petri dish and further drying led to a transparent homogeneous film (Figure 3a), which, however, easily dissolved in water. 

This fact apparently indicated that the IPEC species retained their core–shell structure after deposition on the surface and further drying. The hydrophobic Alg-Pq10 blocks were surrounded by cationic Pq10 fragments, which prevented interparticle contacts between the internal hydrophobic blocks and maintained the solubility of polycomplex particles in water.

The situation changed radically when the coating was made of the IPEC-HR composite. In this case, the initial aqueous formulation was a dispersion (see above) which formed a coating with uniform distribution of medium-sized inclusions (Figure 3b).

**Figure 4:**
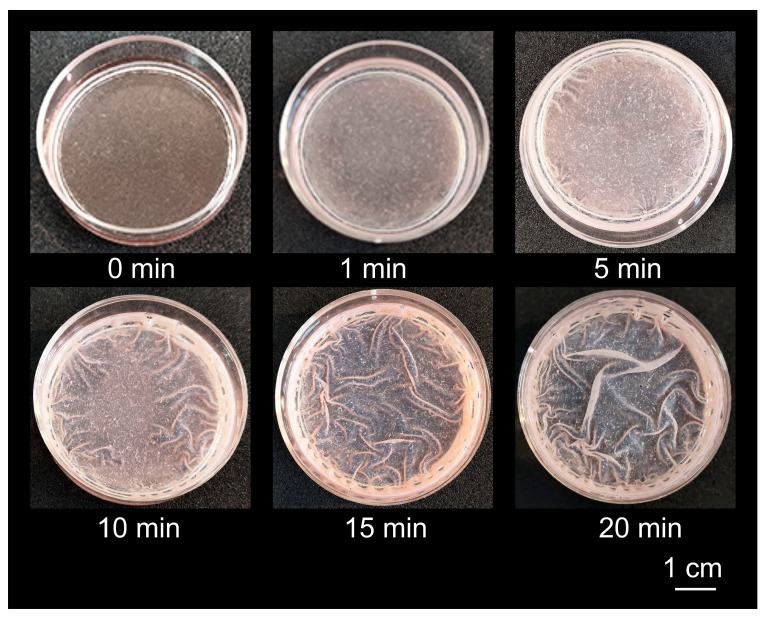
Swelling in water of the coating from IPEC–HR composite pre-formed on a polystyrene Petri dish, as described in the legend to Figure 3b. The scale bar applies to each subfigure.

After the addition of water, the IPEC–HR composite coating only swelled but did not dissolve and did not leave the surface (Figure 4). Higher stability of the composite coating seems to indicate that the composite particles, which also have the core–shell structure, are able to interact with each other via their hydrophobic cores and hydrogen bonds produced by HR molecules. This mechanism allows the composite particles to form a continuous network within the coating and thereby stabilize it.

Moreover, the composite coatings did not leave the surface upon treatment with a 4 N NaCl solution. Under these conditions, the binary IPEC dissociated down to the initial components, Alg and Pq10 [54]. Further drying of the composite sample, covered with the salted solution, resulted in the film, with white NaCl crystals on the top (Figure 3c).

### 2.4. Antimicrobial Activity of IPEC–HR Composite

The next step was to analyze the antimicrobial activity of the IPEC–HR composite in solution and on the surface. In the first case, according to the experimental conditions, the composite formulation was diluted 30 times and more, which resulted in a final ethanol content ˂ 1 wt.%. This had no specific toxic effect on the studied microorganisms [57,58].

The individual polysaccharides, Alg and Pq10, did not show antimicrobial activity up to 1 wt.% polymer concentrations [54], the maximum achieved in the experiment. In other words, both the minimum inhibitory concentrations (MICs) and minimum bactericidal concentrations (MBCs) for both individual polymer values were ˃1 wt.%.

The results of antimicrobial testing for the IPEC–HR composite and HR (control) are summarized in Table 1. The composite formulations showed pronounced antimicrobial activity against Gram-positive bacteria *S. aureus* and *M. luteus*, yeast *Y. lipolytica* and Gram-negative *E. coli*, and lower activity was shown toward Gram-negative *P. aeruginosa*. In most cases, the MIC and MBC values for the composites were comparable with the MIC and MBC values for individual HR. Thus, the incorporation of HR into the Alg-Pq10 IPEC matrix had no negative effect on the HR biocidal activity. 

**Figure 5:**
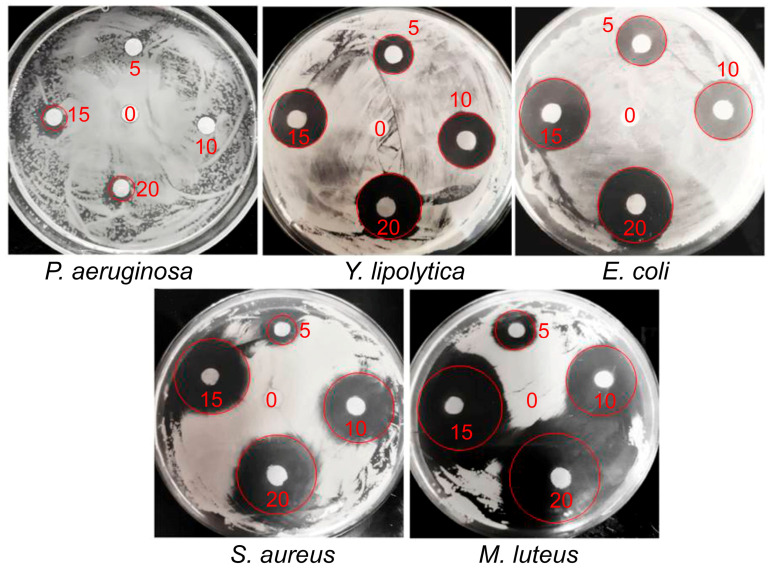
Inhibition zones (red circles) of the IPEC–HR composite against the tested microorganisms; 10^–2^ M pH 7 phosphate buffer/ethanol mixture (98/2 *v*/*v*), Q = 0.2; [Pq10] = 2 × 10^–2^ M; [HR] = 5 × 10^–2^ M. The red numbers on the photos indicate a volume (µL) of IPEC–HR composite applied to the disc. Control sample (20 µL of IPEC without HR) was applied to the central disk and marked as “0”.

The study of antimicrobial activity of the IPEC–HR composite on the surface was quantified using the conventional disk-diffusion method [56,59]. Paper disks were placed on the agar with a uniformly deposited microbial suspension and then different volumes of the composite formulation (from 5 to 20 µL) or 20 µL of HR-free IPEC solution were applied over the disks. This allowed verifying the dose-dependent antimicrobial effect of the composite and comparing it with the effect of the IPEC matrix unloaded with HR. Figure 5 shows the photos of the samples after their incubation at 28 °C for 48 h. The numbers next to the discs indicate the volume of the applied composite or HR-free IPEC.

**Figure 6:**
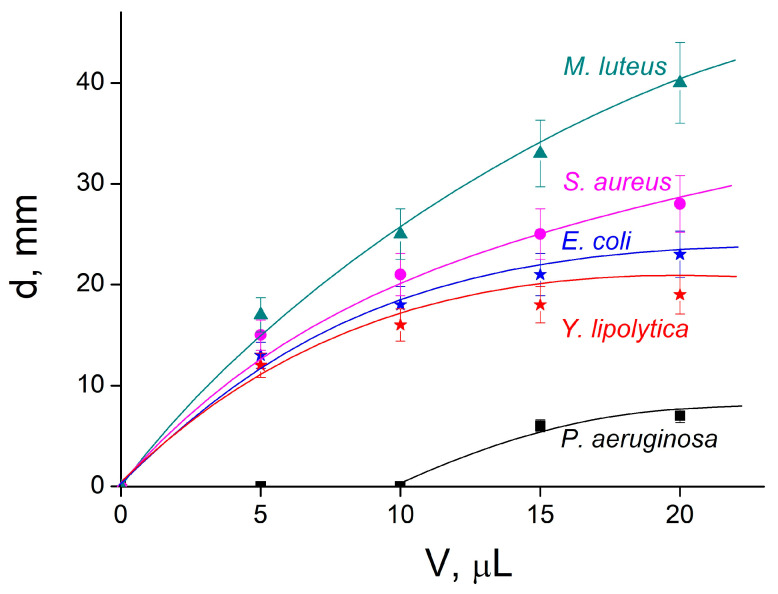
Diameter of inhibition zone from Figure 5 vs. volume of the IPEC–HR composite applied to the disk.

Dark rings around the paper disks reflect microbial growth suppression due to the diffusion of HR from the disks to agar with the microbial culture via a “relay-race” mechanism through repeated formation and the destruction of hydrogen bonds between HR and the agar matrix [56]. The wider the ring, the more active the formulation applied. The dependence of the ring diameter on the volume of applied formulation is given in Figure 6.

As expected, the ring diameter for the HR-free IPEC was equal to zero for all five types of bacteria. When the composite formulation was used, the ring diameter (antimicrobial effect) increased with the elevation of the deposited composite. The most pronounced antimicrobial effect was detected for Gram-positive *M. luteus*, and the lowest was for Gram-negative *P. aeruginosa*. On the whole, the antimicrobial activity of IPEC–HR on the surface, obtained with the use of the disk-diffusion method, correlated well with the MIC/MBC data determined in solution.

## 3. Materials and Methods

### 3.1. Materials

Alg, Pq10, sodium hydroxide, sodium chloride, sodium dihydrogen phosphate monohydrate (all purchased from Sigma-Aldrich Co., St. Louis, MO, USA), 4-hexylresorcinol (HR, Sigma Aldrich, Schnelldorf, Germany), hydrochloric acid and ethanol (purchased from Chimmed, Moscow, Russia) were used without further purification.

All solutions were prepared with double-distilled water additionally filtered through a Milli-Q Millipore system.

Concentrations of the polysaccharides are presented in moles of the ionic groups per liter. The concentration of Alg anionic groups was determined via reverse potentiometric titration [60]. The concentration of cationic quaternary amino fragments of Pq10 was evaluated using the turbidimetric method [61].

### 3.2. Methods

The optical densities of solutions were measured making use of a Genesis™ 50 UV–Visible spectrophotometer (Thermo Fisher Scientific, Madison, WI, USA), using quartz cuvettes with a width of 1 cm.

The pH measurements were taken using a Corning 340 pH meter (Corning Inc., Corning, NY, USA) equipped with a combination glass pH electrode with an integrated temperature sensor.

The electrophoretic mobility (EPM) of particles was measured via laser microelectrophoresis in a thermostatic cell using a Brookhaven Zeta Plus instrument (Holtsville, NY, USA) with the corresponding software.

The mean hydrodynamic diameter of particles was determined via dynamic light scattering at a fixed scattering angle (90°) in a thermostatic cell using a Brookhaven Zeta Plus instrument (Holtsville, NY, USA). Software provided by the manufacturer was employed to calculate diameter values.

The molecular weights (*Mw*) of the polysaccharides were measured using the static light scattering technique at a fixed scattering angle of 90°. The measurements were carried out using a Photocor Complex photometer (Photocor Instruments, Moscow, Russia) equipped with a He–Ne 10 mW laser (λ = 633 nm) as the light source at 25 °C. The results were processed using the Debye equation:

(1) $$\frac{K\times C}{R_{\theta }}=\frac{1}{{\bar{M}}_{w}}+2\times A_{2}\times C,$$

where *K* is the optical constant of the solution, mol × cm^2^ × g^−2^, *C* is the solution concentration, g × cm^−3^, *R_θ_* is the Rayleigh ratio, cm^−1^, and *A*_2_ is the second virial coefficient, mol × cm^3^ × g^−2^ [62].

The refractive index increments of the solutions *∂n/∂c* were measured in a 0.1 N NaCl using an Optilab T-Rex differential refractometer (Wyatt, MO, USA) at λ = 633 nm and 25 °C.

For the light scattering measurements, the solutions were dust cleaned via filtration through a Chromafil^®^ syringe filter (cellulose acetate, pore size of 0.22 μm or 0.45 μm for viscous Pq10 solutions).

Antimicrobial assessments were conducted using five types of microorganisms often used as test cultures: Gram-negative bacteria *Pseudomonas aeruginosa* 4.8.1, *Escherichia coli* MG 1655 K12, Gram-positive bacteria *Staphylococcus aureus* 209P, *Micrococcus luteus* NCIMB 13267 and yeast (eukaryotes) *Yarrowia lipolytica* 367-2 (the microorganism collections of Research Center of Biotechnology RAS). Microorganisms were grown in medium LB (Broth, Miller, VWR Life Science, Radnor, PA, USA) using 250 mL flasks containing 50 mL of nutrient medium. Cultivation was carried out until the stationary growth phase at 28 °C for 1 day using an orbital shaker (120 rpm).

The in vitro antimicrobial activity of the polymer compositions against bacteria and yeast was estimated using the disk-diffusion method [56,59]. The microbial suspensions were applied to Petri dishes containing LB agarized medium (Luria-Bertani Broth, Miller, 1.5% bacteriological agar (Helicon, Moscow, Russia)) and were evenly distributed over the entire surface of the dish using a spatula. Each Petri dish received 5 × 10^7^ to 1 × 10^8^ bacterial or yeast cells. Then, paper disks (Whatman 2017-006 AA Disks 1000 × 6 mm Antibiotic Assay Disks, Whatman, Maidstone, UK-Germany) were placed on the agar, and onto which 5, 10, 15 or 20 µL of the samples were applied. After incubating the dishes in the thermostat at 28 °C for 48 h, the biocidal effect of the polymer formulations was evaluated by measuring the diameter (d, mm) of microbial growth suppression around the discs.

The quantitative assessment of antimicrobial properties of the polymer compositions was carried out via the determination of their minimum inhibitory concentrations (MICs) and minimum bactericidal concentrations (MBCs) [58,63,64,65]. Various aliquots of 1 wt.% polymer solutions were added in the glass test-tubes with 2 mL M9 medium [66,67]. The tubes were inoculated (1% vol) with the microorganisms using 1-day cultures of the stationary growth phase and were placed on a shaker at 28 °C. After 2 days, the growth of the microorganisms was evaluated visually or after sieving on agarized medium.

## 4. Conclusions

Non-stoichiometric ALG-Pq10 IPEC with a 5-fold excess of Pq10 was used for the immobilization of hydrophobic biocidal HR. IPEC particles dispersed in an aqueous solution are capable of absorbing HR up to an [HR]/[Alg] = 10 ratio. After being deposited onto the plastic surface and drying, the IPEC–HR aqueous formulation formed a transparent homogeneous coating, which swelled slightly in water and was retained on the surface even in a 4 N NaCl solution when the water-dispersed IPEC completely dissociated down to Alg and Pq10. The IPEC is substantially non-toxic. The incorporation of HR in the IPEC imparts antimicrobial activity to the resulting composite, in both aqueous solutions and coatings, against Gram-negative and Gram-positive bacteria and yeast. The biocompatible and biodegradable polysaccharide-based IPECs are promising for application in the biomedicine, cosmetology and food industries.

## Figures and Tables

**Table 1:** Antimicrobial activity of HR and IPEC–HR composite.

Tested Microorganism	MIC, wt.%		MBC, wt.%	
	IPEC–HR	HR	IPEC–HR	HR
*P. aeruginosa*	0.035 ± 0.003	0.035 ± 0.002	0.035 ± 0.002	0.035 ± 0.003
*E. coli*	0.0030 ± 0.0002	0.0030 ± 0.0001	0.0050 ± 0.0003	0.0030 ± 0.0002
*Y. lipolytica*	0.0020 ± 0.0001	0.0020 ± 0.0009	˃0.05	˃0.05
*S. aureus*	0.00100 ± 0.00006	0.0020 ± 0.0008	0.0040 ± 0.0003	0.0040 ± 0.0002
*M. luteus*	0.00070 ± 0.00004	0.0020 ± 0.0001	0.0030 ± 0.0001	0.0040 ± 0.0002

## Data Availability

Not applicable.

## References

[1] Zhang L., Xu M., Chen H., Li Y., Chen S. (2022). Globalization, green economy and environmental challenges: State of the art review for practical implications. Front. Environ. Sci..

[2] Mutlu H., Barner L. (2022). Getting the terms right: Green, sustainable, or circular chemistry?. Macromol. Chem. Phys..

[3] Groh K.J., Arp H.P.H., MacLeod M., Wang Z. (2023). Assessing and managing environmental hazards of polymers: Historical development, science advances and policy options. Environ. Sci. Process. Impacts.

[4] Baranwal J., Barse B., Fais A., Delogu G.L., Kumar A. (2022). Biopolymer: A sustainable material for food and medical applications. Polymers.

[5] Rinaudo M. (2008). Main properties and current applications of some polysaccharides as biomaterials. Polym. Int..

[6] Chen T., Sun C., Tian X., Jiang X., Zhang M. (2021). Natural polysaccharide: Modification and application. Pap. Biomater..

[7] Lee K.Y., Mooney D.J. (2012). Alginate: Properties and biomedical applications. Prog. Polym. Sci..

[8] Abasalizadeh F., Moghaddam S.V., Alizadeh E., Akbari E., Kashani E., Fazljou S.M.B., Torbati M., Akbarzadeh A. (2020). Alginate-based hydrogels as drug delivery vehicles in cancer treatment and their applications in wound dressing and 3D bioprinting. J. Biol. Eng..

[9] Marković D., Zarubica A., Stojković N., Vasić M., Cakić M., Nikolić G. (2016). Alginates and similar exopolysaccharides in biomedical application and pharmacy: Controlled delivery of drugs. Adv. Technol..

[10] Azucena Castro-Yobal M., Contreras-Oliva A., Saucedo-Rivalcoba V., Rivera-Armenta J.L., Hernández-Ramírez G., Salinas-Ruiz J., Herrera-Corredor A. (2021). Evaluation of physicochemical properties of film-based alginate for food packing applications. e-Polymers.

[11] Abdullah N.A., Mohamad Z., Khan Z.I., Jusoh M., Zakaria Z.Y., Ngadi N. (2021). Alginate based sustainable films and composites for packaging: A review. Chem. Eng..

[12] Barbut S., Harper B.A. (2019). Dried Ca-alginate films: Effects of glycerol, relative humidity, soy fibers, and carrageenan. LWT.

[13] Senturk Parreidt T., Müller K., Schmid M. (2018). Alginate-based edible films and coatings for food packaging applications. Foods.

[14] Koushki M.R., Azizi M.H., Azizkhani M., Koohy-Kamaly P. (2015). Effect of different formulations on mechanical and physical properties of calcium alginate edible films. J. Food Qual. Hazards Control.

[15] Trevisol T.C., Fritz A.R.M., de Souza S.M.A.G.U., Bierhalz A.C.K., Valle J.A.B. (2019). Alginate and carboxymethyl cellulose in monolayer and bilayer films as wound dressings: Effect of the polymer ratio. J. Appl. Polym. Sci..

[16] Sun J., Tan H. (2013). Alginate-based biomaterials for regenerative medicine applications. Materials.

[17] Ashimova A., Yegorov S., Negmetzhanov B., Hortelano G. (2019). Cell encapsulation within alginate microcapsules: Immunological challenges and outlook. Front. Bioeng. Biotechnol..

[18] Montanucci P., Terenzi S., Santi C., Pennoni I., Bini V., Pescara T., Basta G., Calafiore R. (2015). Insights in behavior of variably formulated alginate-based microcapsules for cell transplantation. BioMed. Res. Int..

[19] Adamiak K., Sionkowska A. (2023). State of innovation in alginate-based materials. Mar. Drugs.

[20] Łętocha A., Miastkowska M., Sikora E. (2022). Preparation and characteristics of alginate microparticles for food, pharmaceutical and cosmetic applications. Polymers.

[21] Terescenco D., Hadj Benali L., Canivet F., Benoit le Gelebart M., Hucher N., Gore E., Picard C. (2021). Bio-sourced polymers in cosmetic emulsions: A hidden potential of the alginates as thickeners and gelling agents. Int. J. Cosmet. Sci..

[22] van der Merwe R.D.T., Goosen N.J., Pott R.W.M. (2022). Macroalgal-derived alginate soil amendments for water retention, nutrient release rate reduction, and soil pH control. Gels.

[23] Borgogna M., Skjåk-Bræk G., Paoletti S., Donati I. (2013). On the initial binding of alginate by calcium ions. The tilted egg-box hypothesis. J. Phys. Chem. B.

[24] Liling G., Di Z., Jiachao X., Xin G., Xiaoting F., Qing Z. (2016). Effects of ionic crosslinking on physical and mechanical properties of alginate mulching films. Carbohydr. Polym..

[25] Deepa B., Abraham E., Pothan L.A., Cordeiro N., Faria M., Thomas S. (2016). Biodegradable nanocomposite films based on sodium alginate and cellulose nanofibrils. Materials.

[26] Hurtado A., Aljabali A.A., Mishra V., Tambuwala M.M., Serrano-Aroca Á. (2022). Alginate: Enhancement strategies for advanced applications. Int. J. Mol. Sci..

[27] Ye J., Ma D., Qin W., Liu Y. (2018). Physical and antibacterial properties of sodium alginate–sodium carboxymethylcellulose films containing Lactococcus lactis. Molecules.

[28] Madzovska-Malagurski I., Vukasinovic-Sekulic M., Kostic D., Levic S. (2016). Towards antimicrobial yet bioactive Cu-alginate hydrogels. Biomed. Mater..

[29] Chiaoprakobkij N., Suwanmajo T., Sanchavanakit N., Phisalaphong M. (2020). Curcumin-loaded bacterial cellulose/alginate/gelatin as a multifunctional biopolymer composite film. Molecules.

[30] Ye Y., Zhang X., Deng X., Hao L., Wang W. (2019). Modification of alginate hydrogel films for delivering hydrophobic kaempferol. J. Nanomater..

[31] Mahcene Z., Khelil A., Hasni S., Akman P.K., Bozkurt F., Birech K., Goudjil M.B., Tornuk F. (2020). Development and characterization of sodium alginate based active edible films incorporated with essential oils of some medicinal plants. Int. J. Biol. Macromol..

[32] Matthews D., Adegoke O., Shephard A. (2020). Bactericidal activity of hexylresorcinol lozenges against oropharyngeal organisms associated with acute sore throat. BMC Res. Notes.

[33] Fidalgo J., Deglesne P.A., Arroya R., Ranneva E., Deprez P. (2018). 4-Hexylresorcinol a new molecule for cosmetic application. J. Biomol. Res. Ther..

[34] Mosangi D., Pillai S.K., Moyo L., Ray S.S. (2016). Inorganic layered double hydroxides as a 4-hexylresorcinol delivery system for topical applications. RSC Adv..

[35] Shariff R., Du Y., Dutta M., Kumar S., Thimmaiah S., Doraiswamy C., Kumari A., Kale V., Nair N., Zhang S. (2022). Superior even skin tone and anti-ageing benefit of a combination of 4-hexylresorcinol and niacinamide. Int. J. Cosmet. Sci..

[36] Stasiuk M., Kozubek A. (2010). Biological activity of phenolic lipids. Cell. Mol. Life Sci..

[37] Kaprelyants A.S., Suleimenov M.K., Sorokina A.D., Deborin G.A., El-Registan G.I., Stoyanovich F.M., Lille Y., Ostrovsky D.N. (1987). Structural-functional changes in bacterial and model membranes induced by phenolic lipids. Biol. Membr..

[38] Tereshkin E.V., Tereshkina K.B., Loiko N.G., Generalova A.A., Kovalenko V.V., Krupyanskii Y.F. (2023). Mechanisms of interaction of Escherichia coli biopolymers with 4-hexylresorcinol. Russ. J. Phys. Chem. B.

[39] Kim S.G. (2022). 4-Hexylresorcinol: Pharmacologic chaperone and its application for wound healing. Maxillofac. Plast. Reconstr. Surg..

[40] Kang Y.J., Jo Y.Y., Kweon H., Kim S.G. (2020). Sericin and 4-hexylresorcinol combination ointment accelerates wound healing in the diabetic burn wound model. Int. J. Indust. Entomol..

[41] Kim J.Y., Seok H. (2020). Role of 4-hexylresorcinol in the field of tissue engineering. Appl. Sci..

[42] Kim S.G., Hahn B.D., Park D.S., Lee Y.C., Choi E.J., Chae W.S., Baek D.H., Choi J.Y. (2011). Aerosol deposition of hydroxyapatite and 4-hexylresorcinol coatings on titanium alloys for dental implants. J. Oral Maxillofac. Surg..

[43] Choo K.W., Dhital R., Mao L., Lin M., Mustapha A. (2021). Development of polyvinyl alcohol/chitosan/modified bacterial nanocellulose films incorporated with 4-hexylresorcinol for food packaging applications. Food Packag. Shelf Life.

[44] Macé S., Hansen L.T., Rupasinghe H.V. (2017). Anti-bacterial activity of phenolic compounds against Streptococcus pyogenes. Medicines.

[45] Wang K., Ye L. (2010). Structure and property of cationic hydroxyethyl cellulose. Polym. Plast. Technol. Eng..

[46] Gao W., Liu X.M., Gross R.A. (2009). Determination of molar mass and solution properties of cationic hydroxyethyl cellulose derivatives by multi-angle laser light scattering with simultaneous refractive index detection. Polym. Int..

[47] Kabanov V.A. (2005). Polyelectrolyte complexes in solution and in bulk. Russ. Chem. Rev..

[48] Van der Gucht J., Spruijt E., Lemmers M., Stuart M.A.C. (2011). Polyelectrolyte complexes: Bulk phases and colloidal systems. J. Colloid. Interface Sci..

[49] Izumrudov V.A. (2008). Self-assembly and molecular ‘recognition’ phenomena in solutions of (bio)polyelectrolyte complexes. Russ. Chem. Rev..

[50] Fertah M., Belfkira A., Taourirte M., Brouillette F. (2017). Extraction and characterization of sodium alginate from Moroccan Laminaria digitata brown seaweed. Arab. J. Chem..

[51] Trivedi M., Branton A., Trivedi D., Nayak G., Singh R., Jana S. (2015). Characterisation of physical, spectral and thermal properties of biofield treated resorcinol. Org. Chem. Curr. Res..

[52] Izumrudov V.A., Mussabayeva B.K., Kassymova Z.S., Klivenko A.N., Orazzhanova L.K. (2019). Interpolyelectrolyte complexes: Advances and prospects of application. Russ. Chem. Rev..

[53] Zezin A.B., Mikheikin S.V., Rogacheva V.B., Zansokhova M.F., Sybachin A.V., Yaroslavov A.A. (2015). Polymeric stabilizers for protection of soil and ground against wind and water erosion. Adv. Colloid Interface Sci..

[54] Novoskoltseva O.A., Belov A.A., Loiko N.G., Nikolaev Y.A., Panova I.G., Yaroslavov A.A. (2022). Biodegradable interpolycomplexes for anti-erosion stabilization of soil and sand. Polymers.

[55] Şen F., Kahraman M.V. (2018). Preparation and characterization of hybrid cationic hydroxyethyl cellulose/sodium alginate polyelectrolyte antimicrobial films. Polym. Adv. Technol..

[56] Kemme M., Heinzel-Wieland R. (2018). Quantitative assessment of antimicrobial activity of PLGA films loaded with 4-hexylresorcinol. J. Funct. Biomater..

[57] Man A., Gâz A.S., Mare A.D., Berţa L. (2017). Effects of low-molecular weight alcohols on bacterial viability. Rev. Romana Med. Lab..

[58] Mazzola P.G., Jozala A.F., Novaes L.C.D.L., Moriel P., Penna T.C.V. (2009). Minimal inhibitory concentration (MIC) determination of disinfectant and/or sterilizing agents. Braz. J. Pharm. Sci..

[59] Bonev B., Hooper J., Parisot J. (2008). Principles of assessing bacterial susceptibility to antibiotics using the agar diffusion method. J. Antimicrob. Chemother..

[60] Novoskoltseva O.A., Panova I.G., Loiko N.G., Nikolaev Y.A., Litmanovich E.A., Yaroslavov A.A. (2021). Polyelectrolytes and polycomplexes for stabilizing sandy grounds. Polym. Sci. Ser. B.

[61] Kabanov V.A., Zezin A.B., Mustafaev M.I., Kasaikin V.A., Goethals E.J. (1980). Soluble interpolymer complexes of polyamines and polyammonium salts. Polymeric Amines and Ammonium Salts.

[62] Litmanovich E.A., Kotova E.V., Efremov V.V. (2019). Dilute-semidilute regime crossover in aqueous solutions of poly (acrylic acid)-sodium poly (styrene sulfonate) mixtures. Colloid. Polym. Sci..

[63] Andrews J.M. (2002). Determination of minimum inhibitory concentrations. J. Antimicrob. Chemother..

[64] Elbing K.L., Brent R. (2019). Recipes and tools for culture of Escherichia coli. Curr. Protoc. Mol. Biol..

[65] Loiko N., Danilova Y., Moiseenko A., Kovalenko V., Tereshkina K., Tutukina M., El-Registan G., Sokolova O., Krupyanskii Y. (2020). Morphological peculiarities of the DNA-protein complexes in starved Escherichia coli cells. PLoS ONE.

[66] Panova I.G., Shevaleva E.A., Gritskova I.A., Loiko N.G., Nikolaev Y.A., Novoskoltseva O.A., Yaroslavov A.A. (2022). Biocidal coatings from complexes of carboxylated latex particles and a linear cationic polymer. Polymers.

[67] Novoskoltseva O.A., Loiko N.G., Nikolaev Y.A., Lisin A.O., Panova I.G., Yaroslavov A.A. (2022). Interpolyelectrolyte complexes based on hydrolyzed polyacrylonitrile for anti-erosion stabilization of soils and ground. Polym. Int..

